# Oral care for intensive care unit patients without mechanical ventilation: protocol for a systematic review and meta-analysis

**DOI:** 10.1186/s13643-021-01878-0

**Published:** 2022-01-05

**Authors:** Xiaoxia Tang, Yunxia Shen, Xuejiao Pan, Jianglong Liao, Yanfei Xu, Wen Luo, Xiaolin Zhang, Chun’e Li, Qing Wan, Xin Cai, Xiaomei Zhang, Tao Wang, Guilan Zhang

**Affiliations:** Kunming Municipal Hospital of Traditional Chinese Medicine, Kunming, Yunnan China

**Keywords:** Oral care, Intensive care unit, Nosocomial pneumonia, Review, Protocol

## Abstract

**Background:**

Infection is a common problem and a major cause of morbidity and mortality for patients in intensive care units (ICUs). According to published meta-analyses, oral care has been found to reduce the risk of nosocomial pneumonia, and has been recommended to improve the oral environment for patients in ICUs. However, relatively little information is available about the effects of oral care in patients without ventilatory support in ICUs. Therefore, this review proposes to evaluate the effectiveness of oral care in preventing pneumonia in non-ventilated ICU patients.

**Methods:**

Eight databases will be searched for relevant literature, including four Chinese and four English online databases, from their inception to the protocol publication date. Records obtained will be managed and screened via Endnote X7. All literature will be selected following pre-established inclusion criteria by two independent review authors to obtain quality trials. The quality of the included records will be evaluated according to the “risk of bias table”, recommended by the Cochrane Handbook for Systematic Reviews of Interventions. All the data will be extracted by one author and checked by another. If there is any disagreement, a final agreement will be reached with a third reviewer via consultation. If there are missing data, the original authors will be emailed to ask for it. If enough data were collected, the data synthesis will be performed using Review Manager (RevMan5.3). Both a random effect model and a fixed effect model will be undertaken. A Bayesian meta-analysis will also be performed to estimate the magnitude of the heterogeneity variance and comparing it with the distribution using the WinBUGS software. Otherwise, the results will be reported narratively. The sources of heterogeneity will be determined using meta-regression and subgroup analysis if there is significant heterogeneity. A funnel plot will be used to assess publication bias if there are enough records included. The Cochrane Handbook for Systematic Reviews of Interventions will be followed throughout the system evaluation process.

**Conclusion:**

This review will provide evidence of oral care for intensive care unit patients without mechanical ventilation to prevent nosocomial pneumonia.

**Trial registration:**

PROSPERO Research registration identifying number: CRD42020146932

## Background

### Description of condition

Infection is a common problem and a major cause of morbidity and mortality for patients in intensive care units (ICUs) [1–4]. It is the leading cause of death in non-cardiac ICUs [1, 5]. Pneumonia is the most common site of infection according to an international study of the prevalence and outcomes of infection in ICUs, which included 13,796 patients [6]. Nosocomial pneumonia (NP) is among the leading causes of mortality in patients in the ICU. Notably, the incidence of nosocomial pneumonia is increasing, and the number of infection-related deaths that follow is also increasing. Thus, preventing nosocomial pneumonia is a cost-reducing and life-saving health care practice, especial in ICUs.

Nosocomial pneumonia (NP), also called hospital-acquired pneumonia (HAP) or healthcare-associated pneumonia, was defined as an infection of the lower respiratory tract that does not exist at the time of admission and does not have an incubation period of infection but occurs 48 hours after admission [7]. The most important cause for the development of nosocomial pneumonia is the oral environment [8]. The oral cavity of ICU patients is an important reservoir for bacteria and provides a habitat for microorganisms that can lead to nosocomial pneumonia [7]. Patients in ICUs acquire pneumonia by aspirating oral bacteria that have been colonized in the oral cavity into the lower respiratory tract. Due to advanced age, limited mobility, illness, and cognitive dysfunction, patients in ICUs often have difficulty maintaining oral hygiene by themselves. Poor dental hygiene has been linked to respiratory pathogen colonization in ICU patients. Therefore, respiratory pathogens tend to colonize dental plaque and oral mucosa in these populations [9]. Therefore, strategies to eliminate respiratory pathogens from the oral cavity may improve oral hygiene and decrease the development of nosocomial pneumonia.

### Description of the intervention

Oral care has been defined as “science and technology for the prevention of oral diseases, improvement of the treatment effect, promotion of the rehabilitation of patients, improvement the quality of life (QOL), and protection of the health” [10]. Oral care has been found to reduce the risk of nosocomial pneumonia according to published meta-analyses [11, 12], and has been recommended to improve the oral environment for patients in ICUs [7]. Oral care aims to remove plaque and debris from the oral cavity. Mouth rinses, including saline, water, and antiseptics may be applied as sprays and liquids during oral care. Antiseptic agent sprays include povidone-iodine, saline, chlorhexidine, cetylpyridium, and possibly others (but exclude antibiotics) [13]. Possible tools include swabs or tooth brushing (manual or powered) which can provide mechanical cleaning [14, 15].

### How the intervention might work

Patients in ICUs often have difficulties completing their oral hygiene due to dysfunction, illness, and old age, which can be responsible for a poor oral environment. Poor oral environment results in the colonization of respiratory pathogens in ICU patients, which is the primary cause of HAP development.

In oral care, manual or electric toothbrushes can be used to provide mechanical cleaning to remove plaque and debris, and replace certain functions of saliva to moisturize and gargle. It can remove plaque on teeth and gum and destroy the biofilm in which plaque bacteria multiply, thereby enhancing oral care.

### Why it is important to do this review

In the past 20 years, most studies have focused on preventing and treating nosocomial infections in mechanically ventilated patients. A number of systematic reviews have focused on the effect of oral care in high-risk patients with ventilator-associated pneumonia (VAP). Oral decontamination with antiseptic [15–18] and antibiotic agents [19] has been reported to significantly reduce VAP risk. However, several recent studies have found that non-ventilator HAP is more common than VAP. Non-ventilator HAP and VAP have similar risk factors and complications. Non-ventilator HAP is associated with a greater overall economic burden [20–22]. Non-ventilator HAP has an underestimated safety issue that seriously affects patient survival. It leads to a significant increase in costs, length of hospital stay and mortality. Prevention and treatment for non-ventilated HAP should be raised to the same level as those with ventilated patients [20]. A clinical study of the compared mortality risk associated with ventilator-acquired bacterial pneumonia and non-ventilator ICU-acquired bacterial pneumonia on the 30-day mortality of ICU patients emphasized the importance of preventing ICU HAP in non-ventilated patients [21].

Although a systematic review and meta-analysis of randomized controlled trials (RCTs) showed a preventive effect of oral care on pneumonia in non-ventilated individuals, this review included patients in hospitals or long-term care facilities, which led to high heterogeneity between the participants [23]. A study was also performed to investigate the preventive effect of oral care for non-VAP older people in nursing homes and hospitals. It included 4 RCTs, in which the results of 3 studies indicated that oral care could effectively reduce the incidence of HAP. However, because of clinical heterogeneity among the 3 studies, meta-analyses were not performed. Another limitation of this review is that the literature searches were conducted only in the Cochrane library and MEDLINE databases. Therefore, there is the potential for existing additional evidence that could be identified when searching beyond these two sources [24]. To address this limitation, this review was designed to evaluate the effectiveness of oral care in preventing pneumonia in non-ventilated ICU patients. The PICOS framework (participants, interventions, comparators, outcomes and studies) was built as follows P: non-ventilated ICU patients; I: oral care; C: non-oral care; O: incidence of nosocomial pneumonia; S: clinical randomized controlled trials. Details are reported in the methods section.

## Methods

### Study design and program registration

This design was registered in the international prospective register of systematic reviews (https://www.crd.york.ac.uk/PROSPERO/ID=CRD42020146932). The details of this protocol adhere to the Preferred Reporting Items for Systematic Reviews and Meta-analyses Protocols (PRISMA-P) statement [25]. Since this review is a secondary study of the literature, formal ethical approval is not applicable.

### Inclusion g criteria

#### Types of studies

Only clinical randomized controlled trials (RCTs) of oral care interventions will be included in this review. The quasi-RCTs and non-RCTs will be excluded. If there is a cross-cover randomized controlled trial, the first period will be included as a parallel-group trial.

#### Types of patients

The ICU patients who did not receive mechanical ventilation without lower respiratory tract infection at admission will be included. HAP was diagnosed as temperature > 37.8 °C, chest radiograph, cough or subjective dyspnoea. For clinical randomized controlled studies involving mechanically ventilated and non-mechanically ventilated patients from ICUs, we will determine whether to include this systematic review based on the proportion of non-mechanically ventilated patients. We will include this review only non-mechanically ventilated patients exceeding 50% of the total number. For clinical randomized controlled studies involving different settings, such as rehabilitation units, nursing homes, and communities, we include in this review only the ones with the number of non-mechanically ventilated patients from the ICUs exceeding 50% of the total number of patients.

#### Interventions

Patients in the experimental group received clearly defined oral care procedures, including decontamination of oropharyngeal cavities with antiseptics, oral and pharyngeal cavity rinses, and nurse-assisted tooth brushing. Patients in the control group received no treatment, ‘usual care’, or placebo. Studies that compared different types of oral care will not be included. Studies in which oral care is used as one part of whole treatment protocols will be excluded.

#### Outcomes

##### Primary outcomes

The incidence of nosocomial pneumonia is defined as the primary outcome of this review. Nosocomial pneumonia was defined as an infection of the lower respiratory tract that is diagnosed at least 48 h after the patient was admitted to the hospital, and was not present or incubated at the time of hospital admission [26].

##### Secondary outcomes

Other outcomes, such as mortality, 30-day mortality, duration of ICU stay, oral health indices (including periodontal index, plaque index, bleeding index, gingival index, aetiological diagnosis results, etc.), the usage of antibiotics, adverse effects of the intervention, and economic data were defined as secondary outcomes. Mortality was defined as all deaths reported in a given population. The 30-day mortality was defined as all deaths reported in a given population within 30 days. The duration of ICU stay was defined as the number of days in the ICU. The periodontal index was defined as a numerical rating scale for classifying the periodontal status of a person or population with a single figure that considers the prevalence and severity of the condition [27]. The aetiological diagnosis results included the number or categories of bacterial colonies in the patients' mouths. The usage of antibiotics includes the type, dosage, frequency, and duration of the antibiotic. The adverse effects include more serious infections, complications, and deaths. The economic data include total hospitalization expenses, nursing expenses, drug expenses, etc.

#### Search strategy

##### Online electronic databases

Four English online electronic databases, Embase (via embase.com), MEDLINE (via PubMed), CINAHL (via EBSCOhost), and Cochrane Central Register of Controlled Trials (CENTRAL), will be systematically searched without language restrictions from their inception to the protocol publication date. Four Chinese-language databases, the WanFang Database, Sino-Med Database, Chinese Science and Technology Periodical (VIP) Database, and China National Knowledge Infrastructure (CNKI) database, will be searched from their inception to the protocol publication date. The English terms were used individually or combined with “intensive care” “nosocomial infection”, “oral care”, and “mouth care”, and the Chinese search terms were “zhong zheng jian hu (intensive care),” “yi yuan huo de xing fei yan (nosocomial infection),” and “kou qiang hu li oral care)”. The search strategy we built for MEDLINE via PubMed is presented in Table 1 after a preliminary search, which was performed according to the Cochrane Handbook for Systematic Reviews of Interventions [28]. Some adaptive changes will be made when searching other databases. Of course, to improve the quality of the research, if the search strategy is fine-tuned or changed during the research, we will explain further in the results report. Before completing the results report, we will conduct a literature search again to avoid missed inspections that affect the reliability of the results.

**Table 1:** Search strategy for MEDLINE via PubMed

No.	Search items
#1	“CRITICAL ILLNESS”[Mesh]
#2	(“critical$” adj5 “ill$”)[tw]
#3	(“depend$” adj5 “patient$”)[tw]
#4	( “INTENSIVE CARE”)[Mesh]
#5	(“intensive care” OR “intensive-care” OR “critical care” OR “critical-care”) [Mesh]
#6	ICU [Mesh] or CCU [tw]
#7	((“intubat$” or “ventilat$”) adj5 “patient$”) [tw]
#8	#1 OR #2 OR #3 OR #4 OR #5 OR #6 OR #7
#9	( “PNEUMONIA, VENTILATOR-ASSOCIATED”)[Mesh]
#10	pneumonia [tw]
#11	VAP [tw]
#12	“nosocomial infection”[tw]
#13	#9 OR #10 OR #11 OR #12
#14	(“ORAL HYGIENE”)[Mesh]
#15	(DENTIFRICES)[Mesh]
#16	(MOUTHWASHES)[Mesh]
#17	(“ANTI-INFECTIVE AGENTS, LOCAL”)[Mesh]
#18	“Cetylpyridinium”[Mesh]
#19	“Chlorhexidine”[Mesh]
#20	“Povidone-Iodine”[Mesh]
#21	(“oral care” OR “mouth care” OR “oral hygien$” OR oral-hygien$ OR “dental hygien$”)[tw]
#22	(“mouthwash$” OR “mouth-wash$” OR “mouth-rins$” OR “mouthrins$” OR “oral rins$” OR “oral-rins$” OR “toothpaste$” OR “dentifrice$” OR “toothbrush$” OR “chlorhexidine$” OR “betadine$” OR “triclosan$” OR “cepacol” OR “Corsodyl” OR “Peridex” OR “Hibident” OR “Prexidine” OR “Parodexor Chlorexil” OR “Peridont” OR “Eludril” OR “Perioxidin” OR “Chlorohex” OR “Savacol” OR “Periogard” OR “Chlorhexamed” OR “Nolvasan” OR “Sebidin” OR “Tubulicid” OR “hibitane”) [tw]
#23	(“antiseptic$” OR “antiinfect$” OR “local microbicide$” OR “topical microbicide$”) [tw]
#24	#14 OR #15 OR #16 OR #17 OR #18 OR #19 OR #20 OR #21 OR #22 OR #23
#25	randomized controlled trial [pt]
#26	controlled clinical trial [pt]
#27	randomized [tiab]
#28	placebo [tiab]
#29	clinical trials as topic [mesh: noexp]
#30	randomly [tiab]
#31	trial [ti]
#32	#25 OR #26 OR #27 OR #28 OR #29 OR #30 OR #31
#33	#8 AND #13 AND #24 AND #32

##### Additional resources

Although there are no full-text articles in Chinese or English, articles with Chinese or English titles or abstracts will also be screened. We will also conduct a hand-search of the reference list of relevant trials, other systematic reviews, and meta-analyses.

### Data collection and analysis

#### Research management and screening

**Fig. 1:**
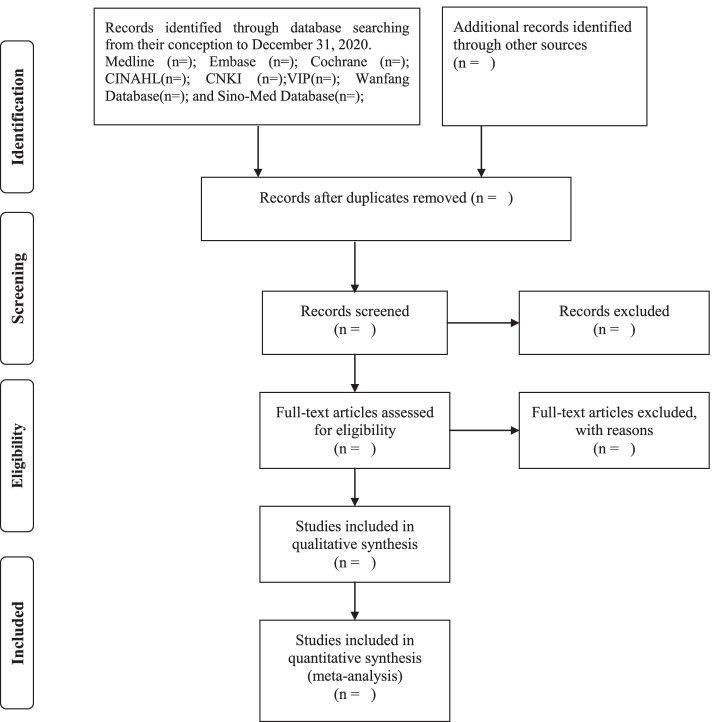
Flow diagram of the study selection process

The literature management software, EndNoteX7, will be used in this review to manage all records collected. Before two independent reviewers read the title and abstract of the trials, duplicate records will be removed from the literature database. Ineligible records will then be identified and removed. The full text of these records will be read independently assessed by two reviewers for potentially eligible records. Finally, eligible literature was selected according to the predetermined inclusion criteria. All records included must fit the type of study, type of patients, intervention, and design (PIS) strategy of this review. The lead author will be contacted via email when more information is needed to decide. If there is any disagreement, the final consensus is generated through discussion with a third reviewer. Details of the literature screening will be reported following the PRISMA flow diagram (Fig. 1). All steps and results of this review will be reported according to the PRISMA 2020 statement [29].

#### Data extraction and management

First, all data will be extracted according to the data table previously prepared by one researcher. Then, all extracted data will be verified by another researcher. The information we will extract is multifaceted. The information will be extracted from each included trial as follows: (1) basic information of the studies, including the journal, year of publication, author, author institutions, and title; (2) characteristics of the participants of RCTs, including sex, age, inclusion/exclusion criteria; (3) intervention used in groups, including the intensity, frequency, and period; (4) methodological information of each trial, including random sequence generation, allocation concealment, blinding of participants, and blinding of outcomes assessment; and (5) outcomes, including instruments, drop-out, follow-up, and adverse events. If there are any missing data, the lead author will be emailed to ask for it. The measurement data will be described using the mean and standard deviation (SD) or standard error (SE). The count data will be described using the number of events. The two arms’ data that most fit this review aims will be extracted rather than all arms if there are more than two arms in the study. Before entering data into RevMan5.3 for analysis, necessary data conversion will be performed via spreadsheet software (Microsoft Excel). If there is any disagreement, a final consensus is generated through discussion with a third review author.

### The risk of bias assessment for included studies

The quality of the included studies will be assessed using version 2 of the Cochrane risk-of-bias tool for randomized trials (RoB2), which was recommended by the Cochrane Handbook for Systematic Reviews of Interventions [28] by two reviewers independently. The domains included in RoB2 are (1) bias arising from the randomization process; (2) bias due to deviations from intended interventions; (3) bias due to missing outcome data; (4) bias in the measurement of the outcome; and (5) bias in the selection of the reported results. For each domain, the toll comprises (1) a series of “signalling questions”; (2) a judgement about risk of bias for the domains; (3) free text boxes to justify responses to the signalling questions and risk-of-bias judgement; and (4) an option to predict (and explain) the likely direction of bias [28]. If there is any disagreement, a final consensus will be generated through discussion with a third review author.

### Handling of missing data

If there are any missing data, original authors will be contacted to request it. If the missing data are not obtained, the analysis will be performed only using the available data. The effect of missing data on the final results will be discussed in the discussion section.

### Assessment of heterogeneity

Heterogeneity across trials will be detected by the *χ*^2^ test with a 0.10 level as the cut-off value (*P* < 0.1). A low *P* value or a large *χ*^2^ statistic provides evidence of heterogeneity of intervention effects. Heterogeneity across trials will be quantified using the *I*^2^ statistic. Studies with an *I*^2^ value of more than 75% will be considered to have a high degree of heterogeneity according to the Cochrane Handbook for Systematic Reviews of Interventions [28]. A Bayesian meta-analysis will also be performed to estimate the magnitude of the heterogeneity variance and comparing it with the distribution suggested by Turner et al. [30] and Rhodes et al. [31] using the WinBUGS software. If the included studies have good homogeneity, the overall effect will be synthesized [32]. Otherwise, the sources of heterogeneity will be explored via subgroup and analysis meta-regression [32]. In addition to statistical heterogeneity, clinical heterogeneity, methodological heterogeneity, and measuring heterogeneity will also be considered in data analysis and result interpretation.

### Evaluation of publication bias

If more than 10 studies report a single outcome, funnel plots will be structured to investigate publication bias [32]. Asymmetry in the funnel plot indicates possible publication bias.

### Data synthesis

If there are sufficient studies focusing on similar comparisons and the same outcomes, both a fixed-effect model and a random-effect model meta-analysis will be undertaken. A Bayesian meta-analysis will also be performed to increase the reliability, credibility, and power of the results suggested by Turner et al. [30] and Rhodes et al. [31] using the WinBUGS software. Otherwise, the results will be narratively reported. The meta-analysis results will be validated using an eight-step procedure according to the report [33]. Additionally, we will apply trial sequential analysis on meta-analyses to adjust for random error risk in meta-analyses. The interpretation of meta-analyses will combine Bayesian meta-analysis with sequential trial analysis, subgroup analyses, funnel plots, meta-regression analyses, etc .[34]

### Subgroup analyses

Clinical heterogeneity, such as whether toothbrushes were used or not and different types of mouthwash, will be considered for subgroup analyses. Subgroup analysis will also be conducted between high-risk and low-risk studies. The results will be reported and discussed in the discussion section.

### Sensitivity analysis

Studies that with a low methodological quality adversely affect the strength of the evidence, so sensitivity analysis will be performed to investigate the effect of these trials on the evidence. It will be performed by excluding a study and comparing the results changes. The results will be reported and discussed in the discussion section.

### Grading the quality of evidence

The quality of evidence for outcomes will be evaluated according to the Grading of Recommendations Assessment, Development and Evaluation (GRADE) system, which rates the quality of evidence into four levels (high, moderate, low, very low levels) [35]. “Summary of findings” tables will be used to present the main findings and key information concerning the quality of evidence via GRADEprofilter and RevMan. “Summary of Findings” tables for each compression included six elements: (1) A list of all important outcomes such as the incidence of nosocomial pneumonia, mortality, 30-day mortality, duration of ICU stay, oral health indices, etc. (2) A measure of the typical burden of these outcomes (e.g., illustrative risk, or illustrative mean, on control intervention). (3) Absolute and (or) relative magnitude of effect. (4) Numbers of patients and studies addressing each outcome. (5) A rating of the overall quality of the evidence for each outcome. (6) Comments.

## Discussion

It was reported that pneumonia is the most common site of infection in ICUs. The occurrence of nosocomial pneumonia is closely related to mortality in ICU patients. The primary mechanism of HAP is the inhalation of bacteria that multiply in the mouth and enter the lower respiratory tract. Patients in ICUs often cannot effectively remove bacteria from their mouths due to illness, older age, or dysfunction. Therefore, helping ICU patients clear bacteria from the mouth effectively can significantly improve their oral environment, thereby reducing the incidence of HAP.

Oral care has been recommended to improve the oral environment. Several studies demonstrated that oral care could significantly reduce the incidence of mechanical VAP. Unlike VAP, the effect of oral care for HAP in ICU patients without ventilatory support has not been well established. Only two reviews focused on the effect of oral care for patients without ventilatory support. However, there was significant clinical heterogeneity between the included studies because they had patients from different institutions, including ICUs, nursing institutions, and rehabilitation settings [23, 36]. Another limitation is the limited database of the sources obtained. Thus, it is necessary to conduct this review to investigate the effect of oral care for HAP in ICU patients without ventilatory support.

We believe that this review results will provide comprehensive and systemic evidence for the effect of oral care for ICU patients without ventilatory support. It will also help make decisions regarding the future practice of oral care for ICU patients without ventilation.

The strengths of the study include rigorous design and a clear definition of participants and intervention. The PRISMA 2020 27-item checklist will be used to improve the quality of results reports [29]. Regardless of the distribution of information priors, Bayesian meta-analysis with information priors can more accurately estimate heterogeneity [31]. Additionally, an eight-step procedure will be used to validate the meta-analysis results, and sequential trial analysis will be used to adjust for random error risk, increase the reliability of the results, and increase the rigor of interpretation of the results. However, there are also some limitations. First, the diversity of oral care media may be one of the sources of clinical heterogeneity. Second, different interventions in the control group between studies may affect the results. Finally, the intervention frequency and operator of oral care may contribute to clinical heterogeneity.

## Data Availability

Not applicable.

## Abbreviations

ICU: Intensive care unit
QOL: Quality of life
HAP: Hospital-acquired pneumonia
NP: Nosocomial pneumonia
RCTs: Randomized controlled trials
VAP: Ventilator-associated pneumonia
CNKI: China National Knowledge Infrastructure
VIP: Chinese Science and Technology Periodical
RoB2: Version 2 of the Cochrane risk-of-bias tool
